# Astrocyte Dysfunctions in Obsessive Compulsive Disorder: Rethinking Neurobiology and Therapeutic Targets

**DOI:** 10.1111/jnc.70092

**Published:** 2025-05-21

**Authors:** Laurine Gonzalez, Paola Bezzi

**Affiliations:** ^1^ Department of Fundamental Neurosciences (DNF) University of Lausanne (UNIL) Lausanne Switzerland; ^2^ Department of Physiology and Pharmacology University of Rome Sapienza Rome Italy

**Keywords:** astrocytes, OCD, psychiatric disorders

## Abstract

Obsessive‐compulsive disorder (OCD) has long been conceptualized as a neuron‐centric disorder of cortico‐striato‐thalamo‐cortical (CSTC) circuit dysregulation. However, a growing body of evidence is now reframing this narrative, placing astrocytes—once relegated to passive support roles—at the center of OCD pathophysiology. Astrocytes are critical regulators of glutamate and GABA homeostasis, calcium signaling, and synaptic plasticity, all of which are disrupted in OCD. Recent high‐resolution molecular and proteomic studies reveal that specific astrocyte subpopulations, including *Crym*‐positive astrocytes, directly shape excitatory/inhibitory balance and control perseverative behaviors by modulating presynaptic inputs from the orbitofrontal cortex. Disruptions in astrocytic neurotransmitter clearance and dopamine metabolism amplify CSTC circuit hyperactivity and reinforce compulsions. This review reframes OCD as a disorder of neuro‐glial dysfunctions, proposing that targeting astrocytic signaling, metabolism, and structural plasticity may unlock transformative therapeutic strategies. By integrating human and animal data, we advocate for a glial‐centric model of OCD that not only enhances mechanistic understanding but also opens new frontiers for precision treatment.

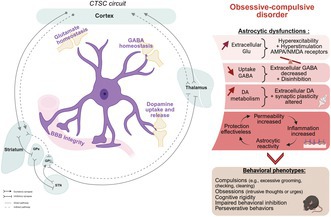

## Graphical abstract

This review highlights a paradigm shift in understanding obsessive‐compulsive disorder (OCD), positioning astrocytes as central players rather than passive support cells. Astrocytes crucially regulate neurotransmitter homeostasis, synaptic plasticity, and neural circuit function within the cortico‐striato‐thalamo‐cortical network, disruptions of which characterize OCD. By outlining specific astrocytic dysfunctions—such as impaired glutamate and dopamine regulation—the review emphasizes their role in OCD pathology and proposes astrocytes as novel therapeutic targets. This glial‐centric perspective not only broadens mechanistic insights into OCD but also paves the way toward innovative treatments for this complex and debilitating psychiatric disorder.

**Abbreviations**
5‐HT: serotonin
ACC: anterior cingulate cortex
AMPA: α‐amino‐3‐hydroxy‐5‐methyl‐4‐isoxazolepropionic acid
APOE3: apolipoprotein E3
BBB: blood–brain barrier
Best1: bestrophin‐1
CNV: copy number variation
COMT: catechol‐o‐methyltransferase
CSF: cerebrospinal fluid
CSTC: cortico‐striato‐thalamo‐cortical
CYPA: cyclophilin A
DA: dopamine
DLGAP1: discs large associated protein 1
DMS: dorsol medial striatum
DRD1/D2/D4: dopamine receptor D1/D2/D4
GAT‐1: GABA transporter 1
GAT‐3: GABA transporter 3
GLAST (EAAT1): glutamate aspartate transporter (excitatory amino acid transporter 1)
GLT‐1 (EAAT2): glutamate transporter 1 (excitatory amino acid transporter 2)
Glu: glutamate
GP: globus pallidus
GRID2: glutamate ionotropic receptor delta type subunit 2
GRIN2B: glutamate ionotropic receptor NMDA type subunit 2B
GWAS: genome‐wide association study
HD: Huntington's disease
IFN‐γ: interferon‐gamma
IL‐6: interleukin‐6
KO: knockout
MAOA: monoamine oxidase A
MAOB: monoamine oxidase B
MMP9: matrix metalloproteinase‐9
MRS: magnetic resonance spectroscopy
NAc: nucleus accumbens
NF‐κB: nuclear factor‐κB
NMDA: N‐methyl‐D‐aspartate
OCD: obsessive‐compulsive disorder
OCT3: organic cation transporter 3
OFC: orbitofrontal cortex
PAPs: perisynaptic astrocytic processes
PFC: prefrontal cortex
SHH: sonic hedgehog
SLC1A1: solute carrier family 1 member 1
SLC6A4: solute carrier family 6 member 4
SSRIs: selective serotonin reuptake inhibitors
ST: striatum
TNF‐α: tumor necrosis factor‐alpha
TSP1: thrombospondin‐1
VEGF: vascular endothelial growth factor
VMAT2: vesicular monoamine transporter 2

## 1 Introduction

Obsessive‐compulsive disorder (OCD) is a chronic and disabling neuropsychiatric disorder characterized by recurrent intrusive thoughts (obsessions) and repetitive behaviors (compulsions), which significantly impact daily functioning and quality of life. Affecting approximately 1%–3% of the global population (Fawcett et al. 2020; Ruscio et al. 2010; Stein et al. 2019), OCD is associated with high rates of comorbidity with anxiety, depression, and related disorders, often emerging in childhood or adolescence and persisting into adulthood (Sharma et al. 2021; Brakoulias et al. 2017). Obsessions manifest as persistent, intrusive, unwanted thoughts, images, or urges and are commonly associated with anxiety (Wilson et al. 2023; Stein et al. 2019; Goodman et al. 2014). Individuals often try to suppress these obsessions or counteract them through compulsive behaviors. Compulsions are repetitive actions or mental rituals that a person feels compelled to perform (Stein et al. 2019; Goodman et al. 2014). These may be in response to an obsession, to adhere to strict self‐imposed rules, or to achieve a sense of completeness. For a diagnosis of OCD, these obsessions and compulsions must significantly impact the individual's life. This impact is typically measured by the time consumed (e.g., taking more than 1 h per day) or by the level of distress and impairment caused in various aspects of life, including personal relationships, social interactions, education, work, and other crucial areas of functioning (Wilson et al. 2023).

### 1.1 Genetic and Environmental Contributions to OCD

Twin studies suggest that genetic factors account for ~40% of the variance in obsessive‐compulsive symptoms, while non‐shared environmental influences contribute ~51%, emphasizing the gene–environment interactions underlying OCD (Pauls et al. 2014; van Grootheest et al. 2005). Several candidate gene studies have implicated polymorphisms in serotonergic (SLC6A4), dopaminergic (COMT, DRD4), and glutamatergic genes (SLC1A1, GRIN2B) in OCD susceptibility, although these studies often lack sufficient power (Pauls et al. 2014).

Genome‐wide association studies (GWAS) confirm that OCD is polygenic, with risk loci of small effect involving glutamatergic genes, including GRID2 and DLGAP1 (Piantadosi et al. 2021). Copy number variation (CNV) studies highlight a 3.3‐fold increased burden of large deletions in neurodevelopmental disorder‐associated regions, including 16p13.11, where de novo deletions are particularly enriched (Piantadosi et al. 2021).

Beyond genetic predisposition, environmental factors are key contributors to OCD onset. Adverse perinatal events, birth complications, and early‐life stress or trauma have been linked to increased risk (Geller et al. 2008). Other studies suggest prenatal infections, maternal immune activation, and environmental toxin exposure may alter neurodevelopmental trajectories, thereby increasing susceptibility to OCD (Wang et al. 2023).

### 1.2 Neural Circuitry and Functional Brain Alterations in OCD

Supporting by neuroimaging studies in patients, OCD is associated with dysfunctions in the cortico‐striato‐thalamo‐cortical (CSTC) circuits, which mediate the balance between goal‐directed and habitual behaviors (Piras et al. 2015; Zhang et al. 2024; Shephard et al. 2021; Burton et al. 2024). This network includes the orbitofrontal cortex (OFC), anterior cingulate cortex (ACC), striatum (ST), thalamus, and prefrontal cortex (PFC), regions involved in cognitive control and response inhibition (Zike et al. 2017; Shephard et al. 2021; Zhang et al. 2024; Calzà et al. 2019; Pauls et al. 2014). Early positron emission tomography (PET) and functional magnetic resonance imaging (fMRI) studies demonstrated increased activation of ventral frontostriatal and temporal regions during symptom provocation (Rotge et al. 2009). This circuitry, which is essential for behavioral control and decision‐making, is now widely recognized as the neuroanatomical basis of OCD (Cerliani et al. 2015; Hou et al. 2014; Jung et al. 2017; Neuner et al. 2014; Tanaka 2021).

Additional studies reveal hyperactivation of fronto‐striatal circuits during executive function tasks such as working memory, response inhibition, and planning, although findings vary based on compensatory recruitment and limbic interference (Gillan et al. 2011; Gillan and Robbins 2014). Notably, similar network abnormalities are observed in first‐degree relatives of OCD patients, supporting a heritable neural endophenotype hypothesis (Gillan and Robbins 2014; Ahmari et al. 2013; Burguière et al. 2013, 2015).

The CSTC pathway is a complex neuronal network connecting the cortex, striatum, and thalamus (Zhang et al. 2024; Shephard et al. 2021; Zhu et al. 2018; Mataix‐Cols and van den Heuvel 2006). The PFC, located in the uppermost part of the frontal lobe, is regarded as the highest integration center for emotional processing and cognitive functions (Carlén 2017). The ST, situated in the brain's core, processes information from other brain regions, particularly from the cortex to the basal ganglia (Hunnicutt et al. 2016). The thalamus acts as a relay for limbic, sensory, and motor information (Sherman 2016; Goodman et al. 2014). In brief, unprocessed signals in these neuronal circuits originate from specific cortical areas, pass through the ST and globus pallidus (where habitual behaviors and conditioned responses are reinforced), then travel through the thalamus (which regulates alertness and relays sensory and motor information) before returning to cortical areas (Zhang et al. 2024). The CSTC circuit comprises two main pathways: direct and indirect (Zhang et al. 2024). The direct pathway, acting as an accelerator, has an excitatory effect on the thalamus via direct projections from the ST to the internal globus pallidus (GPi). The indirect pathway, functioning as a brake, has an inhibitory effect on the thalamus through indirect projections from the striatum to the GPi via the external GP (GPe; Pauls et al. 2014). In healthy individuals, the excitatory direct pathway is modulated by the inhibitory function of the indirect pathway. In OCD patients, an imbalance between these two pathways, with a predominance of the direct pathway, is thought to underlie the manifestations of the disorder (Pauls et al. 2014).

### 1.3 Neurochemical Dysregulation in OCD: The Role of Serotonin, Dopamine, and Glutamate

Dysfunctions in serotonergic (5‐HTergic), dopaminergic (DAergic), and glutamatergic (Gluergic) systems have been strongly implicated in OCD (Fawcett et al. 2020).

#### 1.3.1 Serotonergic Dysfunction in OCD

Selective serotonin reuptake inhibitors (SSRIs) are the first‐line pharmacological treatment for OCD, leading to early hypotheses suggesting that 5‐HTergic deficits play a primary role in the disorder's pathophysiology (Pauls et al. 2014). However, direct evidence supporting a primary serotonin abnormality in OCD remains inconclusive.

While some studies report altered serotonin metabolite levels in cerebrospinal fluid (CSF), which normalize following SSRI treatment, the findings remain inconsistent and lack a clear causal relationship (Wang et al. 2023). Neuroimaging studies have further complicated the 5‐HTergic hypothesis, revealing heterogeneous patterns of serotonin transporter (SERT) and receptor binding across different OCD patient populations (Parmar and Sarkar 2016; Lissemore et al. 2018; Pastre et al. 2025). These inconsistencies suggest that 5‐HTergic abnormalities may represent a compensatory mechanism rather than a primary dysfunction in OCD. Moreover, the therapeutic limitations of SSRIs highlight the complexity of OCD neurobiology. While these medications provide symptom relief for many patients, 30%–40% of individuals exhibit treatment resistance, indicating that other neurotransmitter systems, such as glutamate and dopamine, also play critical roles in OCD pathology (Fineberg et al. 2012, 2013; Stein et al. 2019). The partial efficacy of SSRIs further suggests that 5‐HTergic dysfunction may contribute to, but not fully account for, the compulsive and obsessive features of the disorder.

5‐HTergic receptors play a multifaceted role in the pathophysiology and treatment of OCD. The 5‐HT1B receptor is notably implicated, as its activation disrupts sensorimotor gating and induces repetitive behaviors in animal models (Shanahan et al. 2011; Pittenger et al. 2016). These receptors, densely located in ST and OFC, are critical for modulating such behaviors. Positive correlations between 5‐HT1B receptor binding in the OFC and prepulse inhibition (PPI) have been observed in both controls and OCD patients (Pittenger et al. 2016) and rodent studies (Baldan Ramsey et al. 2011; Swerdlow et al. 2001), while agonists of this receptor increase cFos expression, a marker of cellular activity, in the ST (Ho et al. 2016). These findings suggest that dysfunction of 5‐HT1B receptors contributes to repetitive behaviors seen in OCD.

The 5‐HT2A and 5‐HT2C receptors also play significant roles. Antagonism of the 5‐HT2C receptor enhances reversal learning performance, while antagonism of the 5‐HT2A receptor increases perseverative errors in rodents (Boulougouris et al. 2008; Boulougouris and Robbins 2010). These receptors also influence Gluergic neurotransmission in the OFC, a region hyperactive in OCD patients and rodent models (Saxena and Rauch 2000).

Imaging studies in patients have shown mixed results regarding cortical 5‐HT2A receptor binding in OCD patients (Pastre et al. 2025). Four studies focused on 5‐HT2A (Adams et al. 2005; Perani et al. 2008; Simpson et al. 2011; Wong et al. 2008). In the ACC and the parietal cortex, 5‐HT2AR binding values appeared lower in the OCD group, though these differences lacked statistical significance. Perani et al. hypothesized that this reduction in the cortical region of OCD patients might signal prolonged receptor downregulation caused by insufficient serotonin release (Perani et al. 2008). Conversely, Adam et al. reported higher 5‐HT2A receptor binding in the caudate nucleus in OCD patients' brains (Adams et al. 2005). This study, however, is the only one that demonstrates elevated 5‐HT2A in OCD, and the authors proposed that this finding could reflect a secondary adaptive mechanism, reflecting an increase in receptor density compensating for diminished serotonin levels within the CSTC system. Nonetheless, such results should be interpreted cautiously due to the very low density of 5‐HT2A receptors in the caudate nucleus.

Additionally, serotonin synthesis capacity has been shown to increase globally following successful treatment with SSRIs or cognitive‐behavioral therapy (CBT), indicating enhanced 5‐HTergic tone as a mechanism underlying symptom improvement. However, baseline serotonin synthesis capacity in the raphe nuclei has been positively correlated with treatment response, demonstrating individual variability in serotonergic function. Overall, while receptors such as 5‐HT1B, 5‐HT2A, and 5‐HT2C are clearly involved in OCD pathophysiology, their precise roles require further investigation to better understand their interactions with other neurotransmitter systems and improve treatment strategies.

Given these challenges, a more comprehensive understanding of the serotonergic system's involvement in OCD is required, particularly in its interactions with other neurotransmitter systems and circuit‐level dysfunctions.

Future research should aim to identify biomarkers for SSRI responsiveness and explore novel therapeutic targets beyond serotonin modulation, paving the way for more effective interventions for treatment‐resistant OCD patients (Del Casale et al. 2019).

#### 1.3.2 Dopaminergic Dysfunction and OCD

Dopamine plays a pivotal role in habit formation, reward processing, and compulsive behaviors, and increasing evidence suggests that dopaminergic dysregulation in the striatum contributes to OCD pathology (Zike et al. 2017). Specifically, alterations in DAergic signaling within the dorsal medial striatum (DMS) have been implicated in the reinforcement of compulsive behaviors rather than the overreliance on habitual actions.

Studies employing optogenetic manipulations have provided compelling evidence for this mechanism. Activation of DAergic terminals in the DMS accelerates the onset of compulsive behaviors, while inhibition of these pathways delays their emergence (Seiler et al. 2022). Interestingly, these effects appear to be specific to compulsions rather than habitual behaviors, suggesting that OCD‐related compulsions arise from maladaptive reinforcement of goal‐directed actions rather than from an overreliance on habit learning (Gillan et al. 2011; Voon et al. 2015). Neuroimaging studies in OCD patients further support this hypothesis, revealing reduced striatal dopamine D2 receptor availability, particularly in regions linked to action selection and reinforcement learning (Pauls et al. 2014). This DAergic imbalance may weaken cognitive control mechanisms, allowing intrusive urges to dominate behavior. The therapeutic efficacy of D2 receptor antagonists in a subset of OCD patients underscores the relevance of dopamine dysregulation in the disorder's pathophysiology (Barzilay et al. 2022).

Overall, these findings suggest that aberrant striatal DAergic activity contributes to the persistence of compulsive behaviors in OCD, independent of habitual control mechanisms. This distinction has critical implications for treatment strategies, highlighting the need for interventions that normalize dopamine function while preserving cognitive flexibility in affected individuals.

#### 1.3.3 Glutamatergic Dysregulation: A Central Contributor to OCD

Emerging evidence suggests that glutamate dysfunction is a major driver of OCD pathophysiology, significantly contributing to the hyperactivity observed within CSTC circuits (Piantadosi et al. 2021). Cerebrospinal fluid (CSF) and magnetic resonance spectroscopy (MRS) studies have consistently revealed elevated Gluergic metabolites in OCD patients, indicating excessive excitatory transmission and a failure in glutamate clearance mechanisms (O'Neill et al. 2016; Chen et al. 2019; Naaijen et al. 2015; Pittenger et al. 2011; Wu et al. 2012).

From a genetic perspective, OCD has been linked to multiple Gluergic genes, including SLC1A1, GRIN2B, GRID2, and DLGAP1 (SAPAP3) (Piantadosi et al. 2021). These genes encode critical components of glutamate transport and synaptic function, implicating dysregulation in astrocyte‐mediated glutamate uptake and synaptic Gluergic transmission as key factors in OCD development.

Astrocytes are key regulators of extracellular glutamate homeostasis, and a hallmark of astrocytic dysfunction in OCD is the reduced expression of glutamate transporters, particularly GLT‐1 (EAAT2) and GLAST (EAAT1). This impaired clearance leads to glutamate overflow and elevated extracellular glutamate levels (Aida et al. 2015; Yu et al. 2018; Soto et al. 2023), contributing to hyperactivity within the CSTC circuitry. As a result, NMDA and AMPA receptors in the OFC and ST become overactivated. This sustained excitatory drive reinforces maladaptive synaptic activity and perpetuates compulsive behaviors. These findings support Gluergic dysregulation as a core pathophysiological mechanism in OCD, with astrocyte‐mediated glutamate imbalance playing a central role. Animal models further support this hypothesis, particularly Sapap3 knockout (KO) mice, which lack a critical postsynaptic scaffolding protein involved in cortico‐striatal Gluergic synapses (Welch et al. 2007; Soto et al. 2023; Burguière et al. 2013; Wan et al. 2014). These mice exhibit severe compulsive grooming behaviors, which are alleviated not only by fluoxetine (an SSRI) but also by glutamate‐modulating drugs (Shmelkov et al. 2010). Notably, astrocyte‐specific proteomic analyses in OCD models reveal significant changes in glutamate transporter expression and structural remodeling, further suggesting that astrocytic function is intricately linked to the pathological persistence of compulsions (Soto et al. 2023). Moreover, preclinical studies demonstrate that pharmacologically restoring GLT‐1 function rescues repetitive behaviors in Sapap3 KO mice, reinforcing the notion that targeting astrocytic glutamate regulation may provide novel therapeutic approaches for OCD (Soto et al. 2024). Given the breadth of evidence implicating both neuronal and astrocytic glutamatergic dysfunctions, future research should aim to further elucidate the precise mechanisms by which glutamate homeostasis is disrupted in OCD and explore targeted interventions that restore normal glutamate dynamics in affected circuits.

### 1.4 Astrocytes: The Missing Link in OCD Pathophysiology?

For decades, OCD research has predominantly focused on neuronal dysfunctions within CSTC circuits, emphasizing alterations in neurotransmitter systems such as serotonin, dopamine, and glutamate. However, emerging evidence suggests that astrocytes play a crucial role in maintaining neuronal homeostasis, synaptic function, and neuromodulation, which may have direct implications for OCD pathology (Yu et al. 2018; Ollivier et al. 2024; Soto et al. 2023, 2024; Petrelli et al. 2023). Once considered passive support cells, astrocytes are now recognized as active regulators of synaptic transmission, plasticity, and metabolic signaling (Araque et al. 2014; Kofuji and Araque 2021; Rouach et al. 2008; Escartin and Rouach 2013; Escartin et al. 2021; Soto et al. 2023, 2024; Khakh et al. 2017; Nagai et al. 2021; Santello et al. 2019; Bezzi and Volterra 2001; Pannasch and Rouach 2013; Dallérac and Rouach 2016).

Historically, astrocytes were primarily studied for their role in energy supply and homeostatic maintenance (Bélanger et al. 2011; Magistretti and Allaman 2018; Civenni et al. 1999; Veloz Castillo et al. 2021; Calì et al. 2019; Cantando et al. 2024; Calì 2024). Over the past three decades, their functional complexity has become increasingly evident, revealing their involvement in higher‐order processes such as synapse formation, neuromodulation, neurotransmitter regulation, and neuroimmune signaling (Brazhe et al. 2023; Verkhratsky et al. 2023; Verkhratsky and Nedergaard 2018; Khakh and Deneen 2019; Khakh and Sofroniew 2015; Zehnder et al. 2021; Allen and Eroglu 2017; Allen and Lyons 2018; Farhy‐Tselnicker and Allen 2018). This functional expansion has positioned astrocytes as central elements in neuropsychiatric disorders, including OCD, autism spectrum disorder, and schizophrenia (Allen and Eroglu 2017; Oliveira et al. 2015; Kofuji and Araque 2021; Santello et al. 2019; Banasr and Duman 2008; Lima et al. 2014; Petrelli et al. 2016; de Oliveira Figueiredo, Calì, et al. 2022; de Oliveira Figueiredo, Bondiolotti, et al. 2022; Petrelli et al. 2016; Bezzi et al. 2001; Vesce et al. 1999; Bezzi and Volterra 2011; Petrelli and Bezzi 2018; Ferrucci et al. 2023; Araque et al. 2014). These insights challenge the longstanding neurocentric paradigm, highlighting the necessity of integrating astrocytic dysfunction into the broader pathophysiological framework of OCD.

**FIGURE 1:**
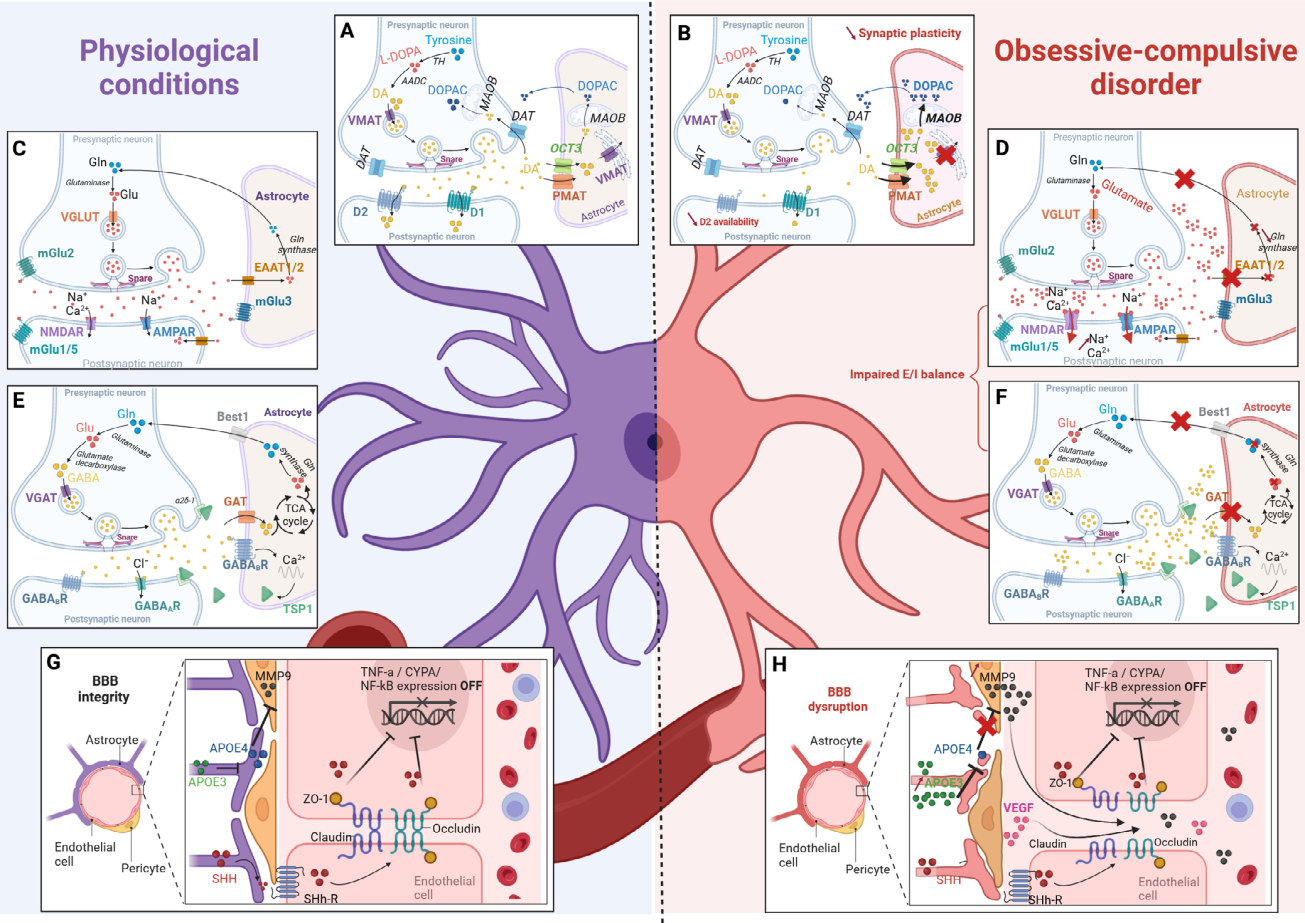
Astrocytic dysfunction disrupts neurotransmitter homeostasis, synaptic plasticity, and blood–brain barrier integrity in OCD. This figure compares physiological (left) and pathological (right) conditions in obsessive–compulsive disorder (OCD), highlighting the central role of astrocytic dysfunction in disrupting neurotransmitter homeostasis, synaptic plasticity, and blood‐brain barrier (BBB) integrity. (A, B) Dopaminergic Neurotransmission. Illustrates dopamine (DA) synthesis from tyrosine in the presynaptic neuron, vesicular transport via VMAT, and release into the synaptic cleft activating postsynaptic D1 and D2 receptors. In physiological conditions (A), DA is cleared from the synapse by PMAT and OCT3 and metabolized by MAOB in astrocytes. In OCD conditions (B), an increase of reuptake of DA by astrocyte, leading to an increase of DA degradation by MAOB, resulting in a decrease of extracellular DA is characterized. AAOD, aromatic amino acid decarboxylase; ADH, aldehyde dehydrogenase; DOPAC, 3,4‐dihydroxyphenylacetic acid; PMAT, plasma membrane monoamine transporterTH, tyrosine hydroxylase. (C, D) Gluergic synapse. Illustrates glutamate (Glu) synthesis from glutamine (Gln) in the presynaptic neuron and vesicular transport via VGLUT. Released Glu activates postsynaptic AMPA and NMDA receptors. In physiological conditions (C), astrocytes clear Glu from the synapse via EAAT1/2. In OCD (D), impaired EAAT1/2 expression leads to increased extracellular glutamate and overstimulation of NMDA and AMPA receptors. (E, F): GABAergic Synapses. GABA synthesis from glutamate in presynaptic neuron and vesicular transport via VGAT. Released GABA activates postsynaptic GABA‐A and GABA‐B receptors. In physiological conditions (E), astrocytes take up GABA via GAT transporters and release it through Best1 channels. In OCD (F), impaired astrocytic GABA uptake leads to decreased GABA‐mediated inhibition. (G, H) Blood–Brain Barrier (BBB) Integrity. The BBB, composed of endothelial cells connected by tight junctions (ZO‐1, Claudin, Occludin), supported by pericytes and astrocyte endfeet. Under physiological conditions (G), the BBB maintains brain homeostasis. The astrocyte releases (Sonic‐Hedgehog) SHH, which inhibits TNF‐α/DIPA/NF‐kB expression in the endothelial cells, leading to BBB integrity and low MMP9 levels. In OCD (H), increased TNF‐α/CYPA/NF‐kB, decreased SHH production, increased MMP9 (matrix metalloproteinase 9), and altered APOE/VEGF contribute to BBB dysfunction and increased permeability. APOE3: apolipoprotein E3; CYPA, cyclophilin; ANF‐κB, nuclear factor‐κB; VEGF, vascular endothelial growth factors. Created in BioRender. Gonzalez, L. (2025).

Several mechanisms through which astrocytes contribute to OCD pathology have been identified (Figure 1):

- Blood–brain barrier (BBB) integrity and neuroinflammation (Figure 1G,H): Astrocytes maintain BBB stability and prevent peripheral immune infiltration. Elevated levels of vascular endothelial growth factor (VEGF) and matrix metalloproteinase‐9 (MMP9), which are markers of BBB dysfunction, have been reported in OCD patients, suggesting a potential neuroimmune component to the disorder (Abbott et al. 2010; Deng et al. 2020).
- Glutamate clearance dysfunction (Figure 1C,D): Astrocytic excitatory amino acid transporters EAAT1 (GLAST) and EAAT2 (GLT‐1) regulate extracellular glutamate levels. In OCD models, a reduction in GLT‐1 expression results in excessive glutamate accumulation, leading to CSTC circuit hyperactivity and reinforcing compulsive behaviors (Soto et al. 2023).
- Dysregulation of GABAergic inhibition (Figure 1E,F): Astrocytes modulate inhibitory neurotransmission via GABA transporters GAT‐1 and GAT‐3. Disruptions in astrocytic GABA uptake lead to an impaired excitation/inhibition (E/I) balance, promoting hyperactivity in CSTC circuits (Nagai et al. 2021; Mederos and Perea 2019).
- Dopaminergic homeostasis and compulsivity (Figure 1A,B): Astrocytes regulate dopamine metabolism through monoamine oxidase B (MAOB) and vesicular monoamine transporter 2 (VMAT2). Studies show that astrocyte‐specific VMAT2 deletion in the PFC induces compulsive behaviors and synaptic pathology, resembling OCD phenotypes (Petrelli et al. 2020, 2023).

### 1.5 Goals of This Review

Given the mounting evidence implicating astrocytes in OCD pathophysiology, this review aims to provide a comprehensive synthesis of their role in the disorder. We will explore the physiological functions of astrocytes in BBB maintenance before and in synaptic regulation, neuromodulation, and examine how their dysfunctions contribute to OCD. By integrating findings from molecular, proteomic, and behavioral studies, we seek to highlight astrocytes as critical players in the neurobiology of OCD. Moreover, we will discuss potential therapeutic avenues targeting astrocyte‐mediated mechanisms, offering new perspectives for the development of effective treatments.

## 2 Astrocytes in Blood–Brain Barrier Function and OCD

Astrocytes are essential regulators of the blood–brain barrier (BBB), a selective structure that protects the central nervous system (CNS) from circulating toxins, pathogens, and peripheral immune cells. Astrocytic end‐feet enwrap endothelial cells and pericytes, forming the glia limitans, a structure that maintains BBB integrity by modulating tight junction proteins and vascular permeability (Abbott et al. 2010; Abbott 2002; Manu et al. 2023). Apart from directly contributing to BBB structure, astrocytes also secrete molecular factors that regulate its stability and permeability. For example, astrocytes actively support BBB integrity by releasing sonic hedgehog (SHH), which strengthens endothelial tight junctions and reduces neuroinflammation (Garcia 2021; Wang et al. 2014; Hill et al. 2021). Another key astrocyte‐derived protein, apolipoprotein E3 (APOE3), plays a protective role by preventing BBB breakdown. APOE3 achieves this by inhibiting a cascade of pro‐inflammatory and degradative signaling pathways, including cyclophilin A (CYPA), nuclear factor‐κB (NF‐κB), and matrix metalloproteinase 9 (MMP9), all of which are known to weaken endothelial junctions and increase vascular permeability (Ferris et al. 2017; Wang et al. 2022; Zhang and Liu 2015; Calì et al. 2024). Conversely, astrocytes can also contribute to BBB disruption through the release of vascular endothelial growth factors (VEGF). While VEGF is essential for angiogenesis and tissue repair, its overproduction in pathological conditions promotes inflammation, increases endothelial permeability, and facilitates the infiltration of immune cells into the CNS (Deng et al. 2020; Abbott 2002). This delicate balance between protective and disruptive astrocytic signals highlights their dual role in BBB regulation and underscores their relevance in neurological and psychiatric disorders.

### 2.1 BBB Dysfunction and Its Implications for OCD

In the context of OCD, emerging evidence suggests that BBB dysfunction may contribute to pathogenesis by altering neuroimmune interactions and exacerbating neuroinflammation. Indeed, elevated levels of VEGF and MMP9, both markers of BBB disruption, have been reported in OCD patients (Abbott 2002; Deng et al. 2020; Abbott et al. 2010). These molecules degrade endothelial tight junction proteins, leading to increased BBB permeability and allowing the infiltration of peripheral immune cells into the brain. This process is particularly significant within cortico‐striatal circuits, which play a pivotal role in compulsive behaviors and OCD pathophysiology (Figure 1G,H).

Astrocytes play a crucial role in modulating BBB integrity, but when exposed to pro‐inflammatory cytokines due to increased BBB permeability, their neuroprotective and homeostatic functions may become impaired. One of the most compelling indications of a neuroimmune‐BBB connection in OCD comes from research on Pediatric Autoimmune Neuropsychiatric Disorders Associated with Streptococcal Infections (PANDAS) and Pediatric Acute‐Onset Neuropsychiatric Syndrome (PANS)—conditions characterized by sudden‐onset OCD symptoms, anxiety, and tics (Swedo et al. 1998, 2022; Chang et al. 2015). In these disorders, it is hypothesized that autoimmune activation and peripheral inflammatory mediators breach the BBB, allowing circulating antibodies and inflammatory cytokines to access the CSTC circuitry (Zhang et al. 2024).

### 2.2 Neuroinflammatory Contributions to OCD and Astrocytic Dysregulation

Elevated levels of pro‐inflammatory cytokines, including interleukin‐6 (IL‐6), tumor necrosis factor‐alpha (TNF‐α), and interferon‐gamma (IFN‐γ), have been reported in OCD patients, further supporting an inflammatory and immune‐mediated contribution to compulsive behaviors (Coelho et al. 2024; Maes et al. 1994; Caldirola et al. 2021; Jose et al. 2021; Karagüzel et al. 2019; Konuk et al. 2007; Westwell‐Roper et al. 2022; Denys et al. 2004). Since inflammatory cytokines are known to modulate BBB permeability (Varatharaj and Galea 2017), these findings suggest that a weakened BBB could serve as a facilitating factor in the neuroimmune alterations observed in OCD (Figure 1G,H).

Astrocytes are highly responsive to inflammatory changes in their environment, and exposure to circulating cytokines can disrupt astrocytic glutamate uptake and gliotransmission (Santello et al. 2011, 2019), leading to excessive excitatory signaling within the CSTC circuit—a hallmark of OCD pathophysiology (Abbott et al. 2010; Abbott 2002). Increased BBB permeability may further expose astrocytes to systemic stressors, such as oxidative stress and microbial metabolites, which could dysregulate astrocytic calcium signaling, impair their ability to buffer extracellular potassium, and promote excitotoxicity in OCD‐relevant brain regions.

Additionally, microglial activation, a common consequence of BBB disruption (Coelho et al. 2024), has been implicated in OCD‐like behaviors in animal models and patients (Coelho et al. 2024; Attwells et al. 2017; Bhattacharyya et al. 2009; Maina et al. 2009; Pearlman et al. 2014; Singer et al. 2015; Dale et al. 2005). Microglia interact with astrocytes to regulate neuroinflammation, and a chronic inflammatory environment can shift astrocytes toward a reactive state, altering their ability to maintain synaptic homeostasis (Kettenmann et al. 2013). Reactive astrocytes in OCD may contribute to excessive glutamate release and impaired inhibitory control, further disrupting the excitation/inhibition (E/I) balance within CSTC circuits (Goubard et al. 2011).

### 2.3 A Perpetuating Cycle: BBB Dysfunction, Neuroinflammation, and Astrocytic Reactivity

This interplay between BBB dysfunction, neuroinflammation, and astrocytic dysregulation may create a self‐reinforcing loop that perpetuates the neural hyperactivity underlying compulsive behaviors. Once BBB integrity is compromised, the infiltration of peripheral immune cells and subsequent inflammatory signaling can push astrocytes into a dysregulated state, impairing their ability to regulate glutamate homeostasis, leading to hyperactivity in CSTC circuits or to maintain GABAergic tone, reducing inhibitory control over compulsive behaviors, or to support dopamine homeostasis, affecting habit formation and reward processing in OCD.

These findings highlight BBB dysfunction as a crucial but underexplored factor in OCD pathophysiology. Given the ability of astrocytes to both maintain and disrupt BBB function, understanding their role in neurovascular integrity may offer novel therapeutic targets aimed at stabilizing astrocyte‐mediated BBB protection and reducing neuroinflammation.

## 3 Astrocytic Regulation of Synaptic Function in Health and OCD

Astrocytes are essential for regulating synaptic activity through their involvement in the tripartite synapse, where they interact with pre‐ and postsynaptic neurons to modulate neurotransmission and synaptic plasticity (Araque et al. 2001, 2014; Bezzi et al. 1998; Sultan et al. 2015; Gómez‐Gonzalo et al. 2018; Calì et al. 2024; Rouach et al. 2008). These functions are mediated by gliotransmission, neurotransmitter uptake, and ion buffering mechanisms, ensuring optimal neuronal communication (Araque et al. 1999; Bezzi and Volterra 2001, 2014; Bezzi et al. 2001, 2004, 1999; Santello et al. 2011, 2012; Petrelli and Bezzi 2016; Vesce et al. 1999; Calì et al. 2009; Cali et al. 2014; Gómez‐Gonzalo et al. 2018; Seifert et al. 2006; Djukic et al. 2007; Calì and Bezzi 2010). By releasing gliotransmitters such as glutamate, D‐serine, and ATP, astrocytes actively shape synaptic activity, modulate plasticity, and regulate the excitatory/inhibitory (E/I) balance, which is fundamental for cognitive functions, learning, and behavior (Araque et al. 2014).

As we will explore in the following sections, compelling evidence suggests that astrocytic dysfunction within the CSTC circuit contributes to the hyperactivity of these neural pathways, a hallmark of OCD pathology (Yu et al. 2018; Aida et al. 2015; Nagai et al. 2021; Olsen et al. 2015). Reduced glutamate uptake, alterations in astrocytic calcium signaling, impaired K+ buffering, and dysfunctional DAergic and GABAergic modulation all converge to create an environment of excessive excitability, reinforcing maladaptive compulsive behaviors and cognitive rigidity characteristic of OCD.

### 3.1 Astrocytes and Glutamatergic Transmission in OCD

Astrocytes play a critical role in maintaining synaptic homeostasis by tightly regulating excitatory glutamatergic transmission, ensuring both efficient synaptic signaling and protection against excitotoxicity. As the primary glial regulators of extracellular glutamate levels, astrocytes express high‐affinity excitatory amino acid transporters, notably EAAT1 (GLAST) and EAAT2 (GLT‐1), which mediate the uptake and clearance of glutamate from the synaptic cleft (Figure 1C; Anderson and Swanson 2000). In fact, the vast majority of glutamate released during neurotransmission is taken up by astrocytes rather than neurons (Papouin et al. 2017), highlighting the essential role of astrocytic clearance in preventing glutamate spillover and excitotoxic damage. Impairments in this mechanism have been implicated in various neuropsychiatric and neurodevelopmental disorders, including OCD, where dysregulated excitatory transmission within CSTC circuits is a hallmark feature of the disease (Figure 1D). Postmortem studies and preclinical models indicate that GLT‐1 expression is significantly reduced in the PFC and ST, resulting in increased extracellular glutamate levels and hyperexcitability (Figure 1D; Aida et al. 2015; Yu et al. 2018; Soto et al. 2023). This disruption is particularly evident in the OFC and ST, regions heavily implicated in OCD pathophysiology. Reduced astrocytic glutamate uptake in these areas may lead to excessive activation of NMDA and AMPA receptors, contributing to repetitive behaviors and impaired cognitive control (Figure 1D).

A significant paradigm shift in OCD research was introduced by Soto et al. (2023), who revealed that astrocytes in the ST are not merely passive support cells but active regulators of neurotransmission and compulsive behaviors. Employing cell‐type‐ and subcompartment‐specific proteomics, their study delineated distinct astrocytic molecular signatures, uncovering widespread dysregulation in OCD‐related circuits. In *SAPAP3* knockout (KO) mice, a well‐established preclinical model of OCD, the absence of astrocytic SAPAP3 disrupted interactions with the glutamate transporter GLT‐1 and the actin‐binding protein Ezrin. This disruption led to reduced astrocyte territory, impaired glutamate homeostasis, and heightened synaptic excitability, culminating in hyperactivation of CSTC circuits. Crucially, comparative analyses between the proteomic alterations in *SAPAP3* KO mice and transcriptomic data from post‐mortem striatal tissue of individuals with OCD revealed overlapping molecular signatures. Notably, shared dysregulated pathways included glutamate signaling, actin cytoskeleton organization, and protein localization processes—each pertinent to SAPAP3's astrocytic functions. These findings underscore the pivotal role of astrocyte–neuron interactions in the pathophysiology of OCD, highlighting potential avenues for therapeutic intervention. Moreover, one of the most striking discoveries was the presence of SAPAP3 in astrocytes, a protein previously believed to be neuron specific. This challenges traditional neurocentric views of OCD, highlighting that astrocytic dysfunction is not merely a secondary consequence of neuronal changes but a primary driver of circuit pathology. Furthermore, their rescue experiments demonstrated that selectively restoring SAPAP3 expression in astrocytes was sufficient to ameliorate OCD‐like compulsive grooming behaviors in *SAPAP3* KO mice.

Recent findings from Soto et al. (2024) provide compelling evidence that astrocytic morphological alterations are central to the pathophysiology of OCD. In *SAPAP3* KO mice, striatal astrocytes exhibited markedly reduced structural complexity, particularly in their fine perisynaptic processes, impairing their capacity to maintain extracellular glutamate and ion homeostasis. These deficits were linked to downregulation of key astrocytic transporters, including GLT‐1 (Slc1a2) and Atp1a2, as well as disrupted actin cytoskeleton organization and G‐protein signaling. Notably, selective activation of the astrocytic Gi‐GPCR pathway using hM4Di DREADDs reversed these morphological and molecular deficits. This intervention restored glutamate and potassium regulation, reduced neuronal hyperexcitability, and significantly alleviated compulsive grooming and anxiety‐like behaviors. Importantly, several of the astrocytic genes and pathways recovered in treated mice overlapped with dysregulated signatures in human OCD post‐mortem striatal tissue, underscoring the translational relevance of astrocyte dysfunction in CSTC circuit dysregulation and OCD pathogenesis. Recent evidence has highlighted the role of intracellular calcium (Ca^2+^) signaling in the onset of perseverative behaviors. Astrocytic Ca^2+^ signaling plays a fundamental role in gliotransmission and synaptic regulation, modulating neurotransmitter release and neuronal excitability. Activation of G‐protein coupled receptors (GPCRs) on astrocytes triggers the phospholipase C (PLC)/inositol 1,4,5‐triphosphate (IP3) pathway, leading to calcium release from intracellular stores (Agulhon et al. 2008; Sobolczyk and Boczek 2022). This Ca^2+^‐dependent mechanism regulates the release of gliotransmitters such as ATP, D‐serine, and glutamate, which in turn modulate neuronal synaptic activity (Di Castro et al. 2011; Bindocci et al. 2017; Bezzi et al. 1998, 2001, 2004; Bezzi and Volterra 2001; Volterra et al. 2014; Domercq et al. 2006; Marchaland et al. 2008; Prada et al. 2011; Petrelli and Bezzi 2016; Buscemi et al. 2017; Cali et al. 2014; Calì et al. 2009; Calì 2024; Parpura et al. 1994; Halassa and Haydon 2010; Sild and Van Horn 2013; Pirttimaki et al. 2017; Volterra and Bezzi 2002). Building on these findings, Yu et al. (2018) demonstrated that selective attenuation of Ca^2+^‐dependent signaling in striatal astrocytes in adult mice induces excessive self‐grooming behavior, a hallmark of OCD‐like phenotypes. This effect is mediated by the upregulation of the astrocytic GABA transporter GAT‐3, leading to a reduction in ambient GABA levels and impaired tonic inhibition of medium spiny neurons (MSNs). The resulting increase in MSN excitability and network synchronization during non‐grooming periods, along with disrupted neuronal activity during grooming episodes, underscores the critical role of astrocytic Ca^2+^ signaling in maintaining striatal circuit balance. Importantly, pharmacological inhibition of GAT‐3 with SNAP5114 normalized both neuronal activity and the repetitive grooming behavior, establishing a causal link between astrocytic Ca^2+^ signaling, neuromodulation, and the control of compulsive behaviors.

In the context of OCD, emerging evidence has identified a specialized population of Crym‐positive striatal astrocytes that gate perseverative behaviors through direct regulation of cortico‐striatal presynaptic terminals (Ollivier et al. 2024). These astrocytes, which account for approximately 50% of striatal astrocytes, modulate the excitatory/inhibitory (E/I) balance of MSNs via a μ‐crystallin‐dependent mechanism—a protein implicated in redox balance, energy metabolism, and thyroid hormone regulation. Loss of Crym expression in astrocytes leads to increased glutamate release from lateral OFC terminals, disrupted synaptic filtering, reduced tonic GABA levels, and the emergence of repetitive behaviors including excessive grooming, marble‐burying, and compulsive licking. Crym‐positive astrocytes contribute GABA to the extracellular space via MAOB and GAT‐3, and their dysfunction results in impaired presynaptic GABAB receptor signaling and elevated glutamatergic drive. These findings highlight the critical role of regional astrocytic heterogeneity in shaping cortico‐striatal circuit dynamics relevant to OCD. Furthermore, they align with previous studies implicating astrocytic GPCR signaling in repetitive behavior (Kofuji and Araque 2021), offering a mechanistic link between astrocytic Ca^2+^ signaling and compulsivity. Gi‐ and Gq‐coupled GPCR pathways in Crym‐astrocytes also influence actin cytoskeleton remodeling, regulating astrocytic morphology and perisynaptic coverage. Disruption of these pathways reduces perisynaptic astrocytic processes (PAPs), limiting astrocytic glutamate uptake and promoting extracellular glutamate accumulation. This exacerbates circuit hyperactivity in the CSTC loop, reinforcing compulsive behavioral patterns.

GPCR activation in astrocytes is known to regulate Ca^2+^‐dependent intracellular signaling, a critical modulator of neuronal excitability and functions. Notably, Ollivier et al. (2024) found that Crym astrocytes display impaired Ca^2+^ signaling, which may alter astrocyte‐neuron interactions within CSTC circuits. Given the importance of astrocytic Ca^2+^ signaling in glutamate uptake and release, a deficit in GPCR‐mediated Ca^2+^ dynamics could result in excessive glutamate spillover, increased NMDA receptor activation, and circuit hyperactivity—mechanisms that are heavily implicated in OCD pathology. Therefore, dysregulation of Crym astrocytes may contribute to excessive glutamate overflow and synaptic hyperexcitability within CSTC circuits. These astrocytes strongly co‐express EAAT1 (GLAST) and EAAT2 (GLT‐1), making them central players in glutamate clearance and synaptic regulation in striatal circuits. Given the established link between reduced astrocytic glutamate transporter expression and OCD‐related hyperactivity (Aida et al. 2015; Yu et al. 2018; Soto et al. 2023), alterations in Crym astrocytes could further exacerbate CSTC dysfunction.

The discovery of Crym astrocytes adds another layer of complexity to our understanding of astrocytic contributions to OCD. By integrating the findings of Ollivier et al. (2024) into our broader model of astrocytic dysfunction in OCD, we gain a more refined perspective on how astrocytes shape compulsive behaviors at the molecular and circuit levels. Future research should focus on how Crym astrocytes respond to therapeutic interventions, including pharmacological targeting of GPCR pathways.

### 3.2 Astrocytes and GABAergic Transmission in OCD

Astrocytes are integral regulators of GABAergic inhibition; a fundamental process required to counterbalance excitatory neurotransmission and maintain CSTC circuit stability. Positioned at inhibitory synapses, astrocytes exert tight control over extracellular GABA levels, shaping synaptic precision and inhibitory tone (Figure 1E). This regulation is predominantly mediated by astrocytic GABA transporters, particularly GAT‐1 and GAT‐3, which remove synaptic GABA and prevent excessive spillover that could lead to aberrant neuronal inhibition (Goubard et al. 2011). While GAT‐1 is distributed on both neuronal terminals and perisynaptic astroglial processes, GAT‐3 is preferentially expressed extrasynaptically, where it regulates inhibitory tone at distal inhibitory sites (Minelli et al. 1996). The coordinated action of these transporters ensures that synaptic inhibition remains spatially confined, preventing the spillover of GABA between neighboring inhibitory synapses while maintaining long‐range inhibitory modulation. Given the crucial role of excitatory‐inhibitory (E/I) balance in neural network function, disruptions in astrocytic GABA regulation have been implicated in neuropsychiatric disorders, including OCD (Figure 1F).

Beyond GABA uptake, astrocytes also actively release GABA, further influencing inhibitory neurotransmission. One major mechanism involves the Best1 (Bestrophin‐1) channel, which facilitates non‐vesicular GABA release, particularly in thalamic circuits (Figure 1E; Lee et al. 2010). This pathway regulates tonic inhibition, modulating ambient GABA levels in the extracellular space and contributing to behavioral control and sensory processing (Yoon et al. 2014; Yoon and Lee 2014; Jo et al. 2014). Additionally, astrocytes can synthesize GABA via monoamine oxidase B (MAOB)‐dependent pathways (Yoon et al. 2014; Lee et al. 2022; Cho et al. 2021), as well as through diamine oxidase and aldehyde dehydrogenase activity. These findings underscore the role of astrocytes as active contributors to GABAergic inhibition, rather than merely passive regulators of synaptic neurotransmitter clearance.

A second key regulatory mechanism is astrocyte‐driven gliotransmission, in which Ca^2+^‐dependent mechanisms modulate GABAergic synaptic activity (Shigetomi et al. 2016; Chai et al. 2017; Yu et al. 2018; Ahrens et al. 2024; Jiang et al. 2016). The activation of GABAB receptors on astrocytes induces intracellular Ca^2+^ oscillations, leading to the release of gliotransmitters such as glutamate and ATP, which modulate synaptic plasticity and inhibitory transmission (Gordon et al. 2009). Importantly, astrocytic GPCR signaling through the PLC/IP3 pathway plays a key role in regulating synaptic activity and behavioral flexibility (Agulhon et al. 2008; Sobolczyk and Boczek 2022).

In the dorsal ST, astrocytic GABAB receptor activation has been shown to promote excitatory synapse formation through thrombospondin (TSP1) signaling, highlighting a role for astrocytes in structural and functional synaptic plasticity (Chung et al. 2013). Similarly, in hippocampal and cortical circuits, GABAB receptor activation on astrocytes triggers Ca^2+^‐dependent glutamate release, which potentiates synaptic activity and behavioral flexibility (Figure 1E; Henneberger et al. 2010; Henneberger et al. 2020). Supporting this, Nagai et al. (2021) uncovered a novel MSN‐to‐astrocyte GABAB signaling pathway that directly regulates astrocytic function, synaptic plasticity, and behavioral hyperactivity. Their study revealed that depolarization of MSNs triggers the release of GABA, which binds to GABAB receptors on neighboring astrocytes, leading to intracellular Ca^2+^ elevations. This activation induced upregulation of TSP1, an astrocyte‐derived synaptogenic cue, which in turn promoted corticostriatal excitatory synapse formation and increased MSN action potential firing. Critically, selective chemogenetic activation of astrocytic Gi‐GPCR pathways in vivo reproduced OCD‐like behavioral phenotypes, including excessive hyperactivity, disrupted attention, and altered synaptic architecture (Figure 1F). These findings suggest that astrocytic GPCR signaling directly contributes to the hyperactive CSTC loops characteristic of OCD, potentially by amplifying excitatory synaptic connectivity through TSP1‐dependent mechanisms. Importantly, the study demonstrated that these astrocyte‐mediated effects were reversible: pharmacological inhibition of TSP1 signaling with gabapentin reversed hyperactivity, synaptic potentiation, and increased neuronal excitability induced by astrocytic Gi‐GPCR activation. This provides compelling evidence that astrocytic GABAB receptor signaling and TSP1‐dependent synaptogenesis play a critical role in CSTC circuit dysfunctions underlying OCD‐related compulsive behaviors (Figure 1F).

### 3.3 Astrocytes and DAergic Dysfunction in OCD

Astrocytes play a crucial role in maintaining dopamine homeostasis through multiple mechanisms, including the uptake, metabolism, and controlled release of dopamine, thereby influencing both synaptic and extrasynaptic dopamine signaling (Figure 1A). They achieve this through the expression of dopamine transporters, MAOB, and the vesicular monoamine transporter 2 (VMAT2), which regulates dopamine storage and availability (Corkrum et al. 2020; Corkrum and Araque 2021; Petrelli et al. 2020). This astrocyte‐mediated modulation of dopamine signaling is essential for motor control and cognitive flexibility, highlighting its relevance in neuropsychiatric disorders such as OCD (Petrelli et al. 2023).

Emerging evidence suggests that dysregulated astrocytic dopamine signaling contributes to the pathophysiology of OCD, particularly by exacerbating hyperactivity within CSTC circuits (Figure 1B; Petrelli et al. 2023). Studies using astrocyte‐specific VMAT2 conditional knockout (aVMAT2cKO) mice show that the selective deletion of VMAT2 in medial PFC astrocytes reduces extracellular dopamine levels, alters synaptic plasticity, and impairs cognitive flexibility—deficits closely resembling neurobehavioral alterations seen in OCD (Figure 1B; Petrelli et al. 2020). Notably, restoring VMAT2 expression in astrocytes or L‐DOPA treatment rescues these deficits, highlighting a direct link between astrocytic dopamine dysfunction and OCD‐like behaviors (Petrelli et al. 2023).

Beyond dopamine depletion, astrocytic dysfunction also leads to maladaptive synaptic plasticity. In aVMAT2cKO mice, corticostriatal synapses exhibit an increased AMPA/NMDA receptor ratio, indicative of excessive excitatory transmission and impaired synaptic filtering, which could reinforce compulsive behavioral loops (Petrelli et al. 2023). These findings align with human neuroimaging studies showing hyperconnectivity between the mPFC and ST in OCD patients, suggesting that astrocyte‐mediated dopamine dysfunction may contribute to the rigid and repetitive behaviors characteristic of the disorder.

Recent work has further demonstrated that astrocytes actively shape DAergic signaling by responding to dopamine via D1 receptors and modulating synaptic transmission (Corkrum et al. 2020; Corkrum and Araque 2021). In the nucleus accumbens (NAc), a key region involved in reward processing and motivation, astrocytes exhibit Ca^2+^ elevations in response to synaptically released dopamine. This activation leads to the release of ATP/adenosine, which suppresses excitatory synaptic transmission via presynaptic A1 receptor activation (Corkrum et al. 2020). Disruptions in this astrocyte‐neuron communication pathway could contribute to the excessive excitatory drive observed in OCD, further supporting the notion that astrocytic dopamine regulation plays a crucial role in behavioral flexibility and inhibition (Corkrum et al. 2020; Corkrum and Araque 2021; Petrelli et al. 2023).

Beyond dopamine uptake and metabolism, astrocytes actively modulate DAergic receptor signaling, further influencing neural excitability and behavioral regulation. Astrocytes express dopamine D1 and D2 receptors, which regulate intracellular Ca^2+^ signaling and gliotransmission (Scofield and Kalivas 2014), thereby shaping synaptic plasticity in response to dopamine input. The role of these receptors in astrocytic function is particularly relevant in the context of OCD, as alterations in D1/D2 receptor balance have been implicated in the development of compulsive behaviors (Scofield and Kalivas 2014). Additionally, astrocytes regulate Gluergic and GABAergic interactions within the CSTC network, contributing to dopamine‐dependent plasticity. Under normal conditions, astrocytes buffer extracellular dopamine, preventing excessive stimulation of D1 receptor‐mediated excitatory pathways. However, in OCD models with astrocytic dopamine dysfunction, reduced dopamine clearance leads to prolonged activation of striatal D1 receptors, excessive excitatory output, and impaired inhibitory control, thereby reinforcing compulsive behaviors (Corkrum et al. 2020; Petrelli et al. 2020, 2023; Corkrum and Araque 2021).

Astrocytes also contribute to the developmental maturation of neuronal circuits, and disruptions in astrocyte‐mediated dopamine regulation during critical neurodevelopmental periods may predispose individuals to OCD and related disorders. VMAT2 expression in astrocytes is dynamically regulated throughout postnatal development, with peak expression occurring during key periods of synaptic pruning and neural circuit refinement (Petrelli et al. 2020). Deficits in astrocytic VMAT2 expression during these periods result in long‐term impairments in dopamine homeostasis, increasing vulnerability to compulsive behaviors later in life. Supporting this, Petrelli et al. (2023) demonstrated that early‐life astrocytic dopamine dysfunction led to persistent alterations in synaptic plasticity and behavioral inflexibility in adulthood. This suggests that disruptions in astrocytic dopamine homeostasis during postnatal development could prime CSTC circuits for hyperactivity, setting the stage for compulsive symptom emergence in adolescence and adulthood. Indeed, early‐life astrocytic dopamine dysfunction can induce long‐term impairments in dopamine homeostasis, increasing vulnerability to compulsive behaviors in adulthood (Barnett et al. 2023). These findings underscore the importance of astrocytic dopamine regulation not only in maintaining synaptic stability but also in shaping the long‐term trajectory of CSTC circuits. OCD‐related deficits in cognitive flexibility and behavioral inhibition may originate from early disruptions in astrocyte‐mediated DA homeostasis, priming CSTC circuits for hyperactivity and contributing to the onset of compulsive symptoms in adolescence and adulthood.

## 4 Implications for OCD Treatment: A Glial‐Centric Perspective

Given the mounting evidence implicating astrocytes in the pathophysiology of OCD, developing astrocyte‐targeted interventions represents a promising avenue for therapeutic innovation. Strategies aimed at restoring Gluergic homeostasis, GABAergic tone, metabolic stability, and synaptic plasticity could help correct the excitatory‐inhibitory (E/I) imbalance and CSTC circuit hyperactivity, which are central to OCD.

### 4.1 Restoring Glutamate Clearance and Preventing Excitotoxicity

Dysregulation of astrocytic glutamate uptake has been consistently linked to OCD pathology, particularly through the downregulation of EAAT1 (GLAST) and EAAT2 (GLT‐1) in PFC and ST. Given that astrocytic GLT‐1 expression is reduced in OCD models, enhancing glutamate clearance may serve as a neuroprotective intervention.

Pharmacological agents such as ceftriaxone, a β‐lactam antibiotic known to upregulate GLT‐1 expression, have shown promise in preclinical models of neuropsychiatric disorders characterized by excessive glutamatergic transmission (Rothstein et al. 2005; Mimura et al. 2011; Miller et al. 2008; Lee et al. 2008; Zhang et al. 2007). Similarly, riluzole (Azbill et al. 2000; Dunlop et al. 2003; Frizzo et al. 2004; Carbone et al. 2012), which enhances astrocytic glutamate uptake and metabolism, has demonstrated efficacy in reducing cortico‐striatal hyperactivity and could be explored as an adjunctive treatment for OCD (Neziroglu et al. 2017). Another potential strategy is to modulate astrocytic GPCR signaling to restore perisynaptic astrocytic processes (PAPs) and improve glutamate homeostasis. Recent findings suggest that activating astrocytic Gαi‐GPCR pathways can promote actin cytoskeleton remodeling, leading to improved astrocytic envelopment of synapses and enhanced glutamate uptake efficiency. Selective DREADD‐based chemogenetic activation of astrocytic Gi‐GPCRs has been shown to normalize synaptic excitability and reduce compulsive‐like behaviors in OCD animal models, indicating its potential for therapeutic translation.

### 4.2 Enhancing GABAergic Function to Restore Inhibitory Control

Astrocytes play a pivotal role in shaping inhibitory transmission, primarily through the regulation of GABA uptake and gliotransmission. Dysregulation of astrocytic GABA transporters (GAT‐1 and GAT‐3) has been observed in OCD models, leading to diminished tonic inhibition and increased neuronal excitability.

Pharmacological interventions aimed at modulating astrocytic GABA regulation could provide a novel approach to correcting inhibitory deficits in OCD. The inhibition of GAT‐3, for instance, has been shown to increase extracellular GABA levels, thereby enhancing tonic inhibition and mitigating circuit hyperactivity. Tiagabine, a GAT‐1 inhibitor, is already FDA‐approved for epilepsy and could be repurposed for reducing compulsive symptoms in OCD by enhancing astrocytic‐mediated GABAergic tone (Oulis et al. 2009). Beyond direct modulation of GABA uptake, targeting astrocytic GABAB receptor signaling may offer additional therapeutic benefits. Nagai et al. (2021) demonstrated that astrocytic GABAB receptor activation promotes thrombospondin‐1 (TSP1)‐mediated synaptogenesis, contributing to hyperconnectivity in CSTC circuits. Gabapentin, which inhibits TSP1 function, successfully reversed OCD‐like behaviors and restored CSTC circuit activity in preclinical models (Onder et al. 2008). This suggests that pharmacological modulation of astrocytic TSP1 signaling may help reduce excessive excitatory synapse formation and normalize CSTC connectivity in OCD.

### 4.3 Targeting Astrocytic Metabolic Dysfunctions in OCD

Recent studies have identified Crym‐positive astrocytes as critical regulators of brain energy metabolism, particularly in high‐energy‐demanding circuits like the CSTC network. These astrocytes exhibit enhanced mitochondrial function, ketone body metabolism, and lipid oxidation, highlighting their role in neuroenergetic homeostasis. In OCD models, metabolic disruptions in Crym astrocytes may contribute to neuronal hyperactivity, reinforcing compulsive behaviors. Therapeutic strategies aimed at enhancing Crym astrocyte function could provide novel metabolic interventions for OCD. Approaches that optimize mitochondrial efficiency, such as mitochondrial uncouplers (e.g., nicotinamide riboside, coenzyme Q10; Chini et al. 2021; Verdin 2015; Harlan et al. 2019; Hathorn et al. 2011; Kishi et al. 2022) or AMPK activators (e.g., metformin, resveratrol; Grassi et al. 2022; Rosso et al. 2016; Tseilikman et al. 2024; Kishi et al. 2022) may help restore astrocytic metabolic flexibility and stabilize neuronal excitability. Furthermore, ketogenic diets, which enhance astrocytic ketone utilization, have been proposed as a potential adjunctive therapy for OCD by reducing neuroenergetic deficits and mitigating CSTC hyperactivity.

### 4.4 Astrocytic Dopamine Regulation as a Target for OCD Therapy

One of the most striking findings in recent research is that astrocytes actively regulate dopamine homeostasis through a complex interplay of VMAT2, OCT3, and MAOB, which are essential for synaptic refinement and executive function (Petrelli et al. 2020, 2023). The selective deletion of astrocytic VMAT2 leads to reduced extracellular dopamine, increased excitatory drive, and compulsive‐like behaviors, strongly resembling OCD pathology. This suggests that restoring astrocytic dopamine buffering capacity through astrocyte‐targeted gene therapy or pharmacological interventions (Agarwal et al. 2008) could mitigate cognitive and behavioral rigidity in OCD patients.

Beyond dopamine metabolism, astrocytes express dopamine receptors (D1 and D2), which regulate intracellular Ca^2+^ dynamics, gliotransmitter release, and dopamine‐dependent plasticity (Corkrum et al. 2020; Araque et al. 2014). Dysregulated astrocytic D1/D2 signaling in OCD could lead to aberrant CSTC excitatory–inhibitory balance, reinforcing compulsive loops. Developing therapies that modulate astrocytic dopamine receptor function could provide more nuanced treatments that do not rely solely on dopaminergic neuronal modulation.

## 5 Future Perspectives

The future of OCD research must increasingly incorporate a glial‐centric perspective, recognizing the active role of astrocytes in synaptic plasticity, neurotransmitter homeostasis, and brain metabolism. While recent studies have shed light on astrocytic dysfunction in OCD, several critical gaps remain that require innovative research strategies to fully understand how astrocytes contribute to the disorder and how they can be targeted therapeutically.

One of the most pressing needs is for longitudinal studies that track astrocytic changes over time in both preclinical models and OCD patients. To date, much of our understanding comes from postmortem analyses or static snapshots of astrocyte function in animal models. Advanced imaging techniques, such as magnetic resonance spectroscopy (MRS) and real‐time astrocyte calcium imaging, could provide invaluable insights into how astrocytic dysfunction evolves at different stages of the disorder. Understanding whether astrocyte pathology is an early driver or a secondary consequence of circuit hyperactivity could help refine therapeutic timing and intervention strategies.

Another key challenge is deciphering the functional and regional heterogeneity of astrocytes within the CSTC circuit. Recent findings, such as those by Soto et al. (2023), reveal that astrocytes are not a homogenous cell population but exhibit molecular and morphological specializations that differentially influence glutamatergic and GABAergic transmission. Future research should focus on mapping the role of distinct astrocyte subpopulations in OCD.

On the therapeutic front, the primary challenge is to develop astrocyte‐selective interventions that go beyond traditional neuron‐targeted treatments. Pharmacological modulation of astrocytic GPCRs, particularly Gαi‐ and Gq‐GPCR pathways, represents a promising avenue. Another promising approach is targeting astrocytic dopamine homeostasis, as studies by Petrelli et al. (2020, 2023) have highlighted the role of astrocytes in buffering extracellular dopamine through VMAT2 and OCT3. Selective enhancement of astrocytic dopamine uptake mechanisms could provide a novel strategy to stabilize dopaminergic signaling in OCD.

Beyond neurotransmitter regulation, metabolic interventions hold significant therapeutic potential. Given that astrocytes are the primary metabolic support cells in the brain, therapies aimed at enhancing astrocytic mitochondrial efficiency, lipid metabolism, or ketone utilization could stabilize neuronal excitability and prevent energy deficits contributing to compulsive behaviors. Dietary interventions (e.g., ketogenic or astrocyte‐specific metabolic modulation) or pharmacological approaches targeting astrocyte‐specific metabolic enzymes could offer new treatment pathways for OCD.

Finally, translational approaches must bridge the gap between basic research and clinical application. Induced pluripotent stem cell (iPSC)‐derived human astrocytes from OCD patients could provide an in vitro model to test astrocyte‐targeted therapies in a personalized manner (D'Antoni et al. 2023). Additionally, gene therapy and optogenetic tools could allow for precise astrocyte modulation in vivo, offering a highly targeted approach to restoring astrocytic function in affected circuits.

In conclusion, the future of OCD research must move beyond a purely neuron‐centric model and embrace the complexity of astrocyte contributions to the disorder. Understanding astrocytic dysfunction in neurotransmitter regulation, synaptic plasticity, and metabolism will not only deepen our knowledge of OCD pathophysiology but also pave the way for next‐generation astrocyte‐targeted therapeutics that address the disorder at its core rather than merely alleviating symptoms.

## Author Contributions

**Laurine Gonzalez:** conceptualization, writing – original draft. **Paola Bezzi:** conceptualization, writing – review and editing.

## Conflicts of Interest

The authors declare no conflicts of interest.

## Data Availability

Data sharing not applicable—no new data generated, or the article describes entirely theoretical research.
